# Emergency department presentations of older patients in Germany: high rates of ambulatory care–sensitive conditions and increased odds of inpatient mortality in patients living with dementia

**DOI:** 10.1186/s12873-025-01458-8

**Published:** 2026-01-06

**Authors:** Kristina Hartl, Anna Slagman, Martin Möckel, Hanna Winkler, Liane Schenk, Thomas Keil, Dorothee Riedlinger

**Affiliations:** 1 Department of Emergency Medicine, Charité – Universitätsmedizin Berlin, Notfall- und Akutmedizin, Notaufnahmen Charité Campus Mitte und Virchow Klinikum, Augustenburger Platz 1, 13,353 Berlin, Germany; 2 OPEN Health, Berlin, Germany; 3 Epidemiology and Health Economics, Institute of Social Medicine, Charité – Universitätsmedizin Berlin, Charitéplatz 1, 10117 Berlin, Germany; 4 Institute of Medical Sociology and Rehabilitation Science, Charité – Universitätsmedizin Berlin; Charitéplatz 1 , 10117 Berlin, Germany; 5 Institute of Clinical Epidemiology and Biometry, University of Würzburg, Würzburg, Germany; 6 State Institute of Health I, Bavarian Health and Food Safety Authority, Erlangen, Germany

**Keywords:** Emergency department, Geriatric emergency care, Dementia, Cross-sectoral healthcare

## Abstract

**Background:**

The crowding of emergency departments (ED) in Germany and many other countries has been a well-known problem and the ageing population is posing additional and very specific challenges to EDs as older patients may present with multimorbidity, polypharmacy, frailty, dementia and non-specific complaints. The primary aim of this study was to characterize clinical and demographic features of emergency department (ED) patients aged ≥70 years in Germany, and to analyse their outpatient care utilization before the ED visit. The secondary aim was to explore the relationship between dementia and inpatient mortality.

**Methods:**

Routine hospital data from 16 German EDs from 2016 were linked to outpatient care data from 2014 to 2017. The demographic and clinical characteristics of ED patients ≥70 years were evaluated for the four billing quarters preceding the first ED visit in 2016. The relationship between dementia and inpatient mortality was assessed using a directed acyclic graph and a generalized linear mixed model adjusted for confounders (e.g., age, sex, comorbidities) and ED centre.

**Results:**

In 2016, 99,858 patients aged ≥ 70 years presented to one of the 16 EDs. Most arrived via medical transportation (60.5%). Whilst 31.2% were triaged as less urgent, 64.2% were admitted as an inpatient. Among the most common ED diagnoses were several ambulatory care–sensitive conditions (ACSC). Although 84.1% of patients having had regular contact (i.e., in ≥ 3 billing quarters) with a general practitioner, geriatric assessment was only conducted in 45.7% of patients; 60.2% of ED patients were taking five or more medications (polypharmacy). ED patients with dementia (*n* = 14,511) had increased odds of inpatient mortality (adjusted odds ratio 1.18; 95%-confidence interval 1.08–1.28).

**Conclusion:**

The frequent occurrence of ACSC and non-urgent conditions in the ED highlights the need for adequate primary healthcare services for acute medical conditions in Germany, particularly for older patients.

**Supplementary Information:**

The online version contains supplementary material available at 10.1186/s12873-025-01458-8.

## Introduction

The World Health Organization estimates that between 2020 and 2050, the number of individuals aged ≥60 years will double and the number of those aged ≥80 years will triple worldwide [1]. An ageing population significantly impacts healthcare systems in terms of resource use and costs as older individuals use healthcare services more frequently than younger individuals [2]. The crowding of emergency departments (ED) in Germany and many other countries has been a well-known problem for years [3, 4]. The underlying reasons are manifold, some of them internal to the hospital, like shortages in ED staff and delayed transfer of patients to an inpatient bed [4]. External causes that substantially contribute to ED crowding include access barriers and insufficient primary care structures for acute but non-emergency cases and inadequate continuity of primary care, and a sizeable population of patients who have urgent, complex conditions and older age [4]. In addition, access to EDs is not regulated in Germany and patients can present to the ED without a prior primary care visit. ED visits of older patients are associated with many challenges as they may present with multimorbidity, polypharmacy, frailty and non-specific complaints which make it difficult to distinguish an urgent problem from existing health issues [5]. Patients in which these effects are even more pronounced are older individuals living with dementia. This particularly vulnerable population requires a high level of healthcare, is of older age (≥ 85 years), presents with a higher comorbidity burden, and in addition, shows higher rates of hospitalization and in-hospital mortality compared with older patients without dementia [6–10].

Data on ED visits and prior outpatient care utilization are scarce in Germany and the strict separation of the primary and secondary care sectors poses additional challenges for cross-sectoral data linkage and analysis. However, it is crucial to know the features, healthcare trajectories and clinical outcomes of older patients to adapt emergency healthcare to the specific needs of older patients to improve cross-sectoral care and patient outcomes.

Therefore, the primary objectives of this study are to describe the demographic and clinical characteristics of patients ≥70 years who presented to EDs in Germany in 2016 and to analyse their outpatient care utilization in terms of the frequency of their general practitioner (GP) and specialist visits in the year before their ED presentation. Since older patients with dementia are reported to have worse clinical outcomes, the secondary objective of this study is to explore the causal relationship between dementia and inpatient mortality following an ED visit.

## Methods

### Data source

This study is a sub-analysis of the INDEED study (utilization and cross-sectoral patterns of care for patients admitted to emergency departments in Germany) which was described in detail by Fischer-Rosinský et al. (2021) [11]. In brief, INDEED collected routine clinical data from 16 EDs across Germany between 1st January and 31st December 2016 and linked these data with outpatient care data from the German Statutory Health Insurance (SHI) Physicians Associations from 1st January 2014 to 31st December 2017 [11]. Thus, SHI outpatient care data were available for the two years prior to and the year after the ED visits in 2016 [11]. The SHI outpatient care dataset included information on outpatient diagnoses based on International Classification of Diseases 10th Revision (ICD-10) codes, healthcare services based on billing codes and data on prescription medication based on Anatomical Therapeutic Chemical Classification codes (ATC codes). The INDEED dataset included adults aged 20 years or older on 1st January 2016 who had at least one ED visit in one of the participating centres in 2016 and who were insured with one of the German SHI companies [11]. ED cases with statutory health insurances account for 80–85% of all ED cases in Germany [12, 13]. The INDEED data set does not contain ED cases with private insurance or those billed via the German Social Accident Insurance [11]. The INDEED study was registered at the German registry for clinical trials (DRKS00022969) on 22.10.2020.

### Study populations

The study sample included all patients aged ≥70 years from the INDEED dataset, stratified into older (70–84 years) and very old (≥ 85 years) subpopulations according to common classifications in the literature.

Two different analyses were carried out: a case-based analysis – in which an individual patient may represent several cases – to describe the burden of ED utilization from the hospital perspective, and a person-based analysis to report healthcare trajectories in terms of outpatient care utilization prior to ED visits.

For the patient-based analysis, the first ED visit was defined as the index visit and patients were grouped according to their age at the index visit. Only ED patients for whom SHI claims data were available, were included in the outpatient population. According to the M2Q (at least two quarters) criterion, which is widely used in the German SHI to validate chronic diagnoses [14], chronic conditions were assumed if a specific diagnosis was billed in two or more different billing quarters within the four quarters preceding the ED visit. All patients from EDs with incomplete SHI data were excluded from the outpatient and chronic diagnosis populations.

Older patients living with dementia were defined as individuals who had a chronic diagnosis of dementia according to ICD-10 codes F00, F01, F02, F03, or G30.

### Descriptive variables

Detailed definitions of all descriptive ED and outpatient variables that were used in the analyses are provided in Table S1 including the number of missing values for the person-based and case-based analyses, respectively. The outpatient care utilization analysis was limited to the four billing quarters preceding the index ED visit of a patient. M2Q-validated, three-digit ICD-10 codes were used to calculate the Charlson Comorbidity Index (CCI) scores according to Quan et al. (2011) [15], and to identify the most common chronic diagnoses of the study populations. In the German SHI system, outpatient healthcare services are defined, recorded and billed via the SHI doctor’s fee scale which includes a specific billing code for each outpatient service (“Gebührenordnungsposition”, GOP). The INDEED dataset contained GOP data including the date and type of outpatient services, and information on the healthcare provider who billed the GOP, e.g., their medical specialization. To assess type and frequency of outpatient care utilization, the number and percentage of patients were calculated who at least once presented to any type of physician within the four billing quarters before the ED visit, who at least once visited the GP and who visited the GP on a regular basis, i.e., in each of the four billing quarters preceding the ED visit. In addition, the most frequently visited specialists were extracted. GOPs were analysed to assess the use of geriatric assessment by the GP. The billing of GOPs indicating geriatric assessment – which can be billed from the age of 70 years – was used as an indicator for the prevalence of frailty and geriatric conditions in this study and was also considered a quality indicator of healthcare delivery for older patients in the primary care sector. Geriatric assessment procedures are only billable for patients who present with specific geriatric morbidities like mobility disorders, cognitive impairment, frailty syndrome, incontinence or dysphagia [16]. The relevant GOPs were: GOP 03360 ‘basic geriatric assessment’, GOP 30,984 ‘extended geriatric assessment’, and GOP 03362 ‘further geriatric care services’. The latter can only be billed if a prior geriatric assessment based on GOP 03360 or 30,984 was conducted. The analysis of prescription medications was limited to the two billing quarters preceding the index ED visit as this was considered a relevant timeframe to detect potential correlations between specific prescriptions and their effect on ED visits. Data on prescription medication were only included if a date of dispensing from the pharmacy was reported to approximate medication intake. ATC codes were used to identify the most frequently prescribed medications and to identify patients with polypharmacy, defined as receiving ≥5 prescription medications.

### Directed acyclic graph and generalized linear mixed model

A targeted literature search was conducted in PubMed, Google and Google Scholar to identify variables which were causally related to an increased risk for the exposure dementia and the outcome death. Subsequently, a directed acyclic graph (DAG) was developed in dagitty to identify and visually represent confounders and causal relationships for adjusting the statistical model [17]. Based on the DAG, a generalized linear mixed model was fitted, using a complete case analysis, and adjusted for confounding and study centre as random effect. Supplementary Figure S1 shows the identified variables and their relationship to both the exposure “dementia” and the outcome “death” as confounders, no confounders, mediators or unobserved variables. Confounding variables represented in the INDEED dataset were hypertension, obesity, depression, diabetes, age, atrial fibrillation and atherosclerosis. Moreover, the INDEED variables GP visits, geriatric assessment, and polypharmacy were identified to be mediators in the model. The outcome variable and all confounding variables except age were modelled as binary variables. Age was included as a continuous variable in the model.

### Statistical analysis

All analyses were performed using R version 4.3.1 [18]. Descriptive statistics were applied to characterize the demographic, clinical and outpatient care utilization features of the ED population. Age was reported as median and interquartile range (IQR). The absolute and relative frequencies of all other descriptive variables were reported as counts and percentages. The model results were reported as odds ratios (OR) and 95% confidence intervals (CI). The threshold of statistically significant differences between groups was set at 0.05. Missing values were assumed to be missing completely at random and thus, no imputations were conducted.

### Ethics and data protection

The ethics committee of the Charité – Universitätsmedizin Berlin approved of the INDEED study (application: EA4/086/17). In accordance with both institutional requirements and national legislation no written informed consent was required from participants. Study procedures followed the principals of the Declaration of Helsinki. The Technology, Methods, and Infrastructure for Networked Medical Research (TMF) working group of data protection and the institutional data protection officer at Charité – Universitätsmedizin Berlin gave their approval for INDEED in February 2018. Further details on ethics and data protection of the INDEED study were reported by Fischer-Rosinský et al. (2021) [11].

## Results

### Size of the study populations

**Fig. 1:**
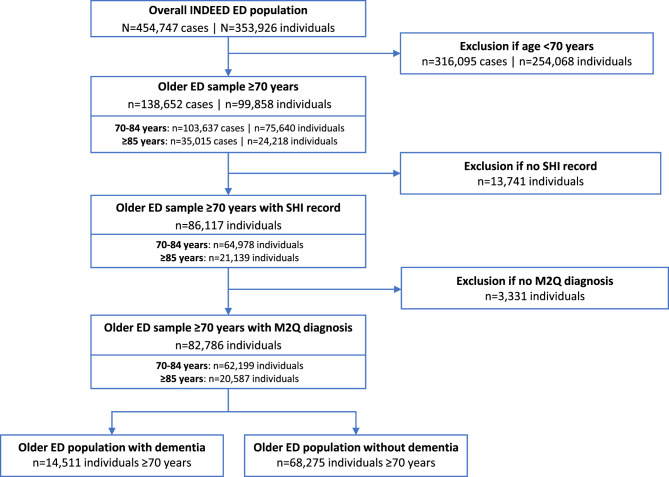
Flow chart of study patients. Abbreviations: ED, emergency department; INDEED, utilization and cross-sectoral patterns of care for patients admitted to emergency departments in Germany; SHI, Statutory Health Insurance

A total of 138,652 ED cases representing 99,858 individual ED patients were aged ≥70 years, which corresponded to 30.5% of cases (visits) and 28.2% of patients of the overall INDEED population. Of the ED population aged ≥70 years, 75,640 patients (75.7%) were aged 70–84 years (representing 74.7% of all visits), and 24,218 patients (24.3%) were ≥85 years (representing 25.3% of all visits). SHI records (outpatient population) and chronic diagnoses (chronic diagnosis population) were available for 86,117 (86.3%) and 82,786 (82.9%) patients, respectively. A total of 14,511 patients (14.5%) had a diagnosis of dementia. An overview of the data selection process and the analysed sub-populations is provided in the flow chart in Fig. 1.

### Demographic and clinical characteristics

In Table 1, patient characteristics for the overall older ED population, age subgroups, patients with chronic diagnoses and patients with and without dementia are provided. With 55.8%, slightly more ED patients ≥70 years were female. Two-thirds of the patients were categorized as urgent according to the Manchester Triage System/Emergency Severity Index. Almost half (45.0%) of patients ≥70 years presented to the ED outside core working hours, i.e., between 19:00 pm and 6:59 am, and over a quarter (28%) during the weekend or public holidays. Demographic and clinical characteristics of the ED case-based analysis are provided in Supplement Table S2.

Patients with dementia were older with a median age of 83 years (IQR, 78–88) versus a median of 78 years (IQR, 75–83) in patients without dementia. A total of 81.7% of the dementia population arrived medically accompanied at the ED versus 59.1% in the population without dementia. Inpatient death occurred more often in patients with dementia with 8.2% versus 6.1% in patients without dementia (Table 1).

**Table 1:** Demographic and clinical characteristics of emergency department (ED) patients, stratified by age group and dementia diagnosis

Characteristics	Overall ED population (*n* = 99,858)			ED population with chronic diagnoses (*n* = 82,786)		
	Patients ≥ 70 years *n* = 99,858	70–84 years *n* = 75,640 (75.7%)	≥ 85 years *n* = 24,218 (24.3%)	Patients ≥ 70 years *n* = 82,786	No dementia diagnosis *n* = 68,275 (68.4%)	With dementia *n* = 14,511 (14.5%)
Age, median (Interquartile range)	79 (75;84)	77 (74;80)	88 (86;91)	79 (75;85)	78 (75;83)	83 (78;88)
Female, n (%)	55,760 (55.8%)	39,481 (52.2%)	16,279 (67.2%)	46,906 (56.7%)	37,914 (55.5%)	8,992 (62.0%)
Transport to ED						
By own means, n (%)	22,499 (31.7%)	19,248 (35.9%)	3,251 (18.8%)	17,324 (29.3%)	15,953 (32.8%)	1,371 (13.0%)
Medically accompanied^a^, n (%)	42,958 (60.5%)	29,924 (55.8%)	13,034 (75.3%)	37,365 (63.1%)	28,766 (59.1%)	8,599 (81.7%)
Other, n (%)	5,529 (7.8%)	4,501 (8.4%)	1,028 (5.9%)	4,497 (7.6%)	3,945 (8.1%)	552 (5.2%)
Missing values, n	28,872	21,967	6,905	23,600	19,611	3,989
Triage category						
Urgent^b^, n (%)	54,055 (68.1%)	40,820 (67.7%)	13,235 (69.5%)	45,304 (69.5%)	37,324 (69.5%)	7,980 (69.5%)
Less urgent^c^, n (%)	25,268 (31.9%)	19,449 (32.3%)	5,819 (30.5%)	19,918 (30.5%)	16,410 (30.5%)	3,508 (30.5%)
Missing values, n	20,535	15,371	5,164	17,564	14,541	3,023
Time of ED admission						
7:00 am – 18:59 pm, n (%)	54,907 (55.2%)	41,709 (55.3%)	13,198 (54.7%)	45,886 (55.4%)	38,133 (55.9%)	7,753 (53.4%)
19:00 pm – 6:59 am, n (%)	44,650 (44.8%)	33,698 (44.7%)	10,952 (45.3%)	36,900 (44.6%)	30,142 (44.1%)	6,758 (46.6%)
Missing values, n	301	233	68	NA	NA	NA
Day of ED admission						
Weekday, n (%)	71,579 (71.9%)	54,235 (71.9%)	17,344 (71.8%)	59,651 (72.1%)	49,382 (72.3%)	10,269 (70.8%)
Weekend/public holiday, n (%)	27,978 (28.1%)	21,172 (28.1%)	6,806 (28.2%)	23,135 (27.9%)	18,893 (27.7%)	4,242 (29.2%)
Missing values, n	301	233	68	NA	NA	NA
Season of ED admission						
Spring, n (%)	26,546 (26.7%)	20,165 (26.7%)	6,381 (26.4%)	22,168 (26.8%)	18,332 (26.8%)	3,836 (26.4%)
Summer, n (%)	22,991 (23.1%)	17,664 (23.4%)	5,327 (22.1%)	18,959 (22.9%)	15,777 (23.1%)	3,182 (21.9%)
Autumn, n (%)	21,016 (21.1%)	15,900 (21.1%)	5,116 (21.2%)	17,268 (20.9%)	14,322 (21.0%)	2,946 (20.3%)
Winter, n (%)	29,004 (29.1%)	21,678 (28.7%)	7,326 (30.3%)	24,391 (29.5%)	19,844 (29.1%)	4,547 (31.3%)
Missing values, n	301	233	68	NA	NA	NA
Type of admission						
Ambulatory, n (%)	35,758 (35.8%)	28,065 (37.1%)	7,693 (31.8%)	28,316 (34.25%)	23,369 (34.2%)	4,947 (34.1%)
Inpatient, n (%)	64,100 (64.2%)	47,575 (62.9%)	16,525 (68.2%)	54,470 (65.8%)	44,906 (65.8%)	9,564 (65.9%)
Mortality						
Inpatient death, n (%)	4,187 (5.3%)	2,613 (4.4%)	1,574 (7.9%)	3,458 (5.4%)	2,684 (5.1%)	774 (6.9%)
Missing values, n	20,872	16,460	4,412	18,632	15,411	3,221
Frequency of ED admission						
1-2x per year, n (%)	91,687 (91.8%)	69,773 (92.2%)	21,914 (90.5%)	75,784 (91.5%)	62,856 (92.1%)	12,928 (89.1%)
3-9x per year, n (%)	8,087 (8.1%)	5,803 (7.7%)	2,284 (9.4%)	6,925 (8.4%)	5,361 (7.9%)	1,564 (10.8%)
> 9x per year, n (%)	84 (0.1%)	64 (0.1%)	20 (0.1%)	68 (0.1%)	49 (0.1%)	19 (0.1%)

^a^ ambulance, emergency ambulance, intensive care transport vehicle/helicopter, rescue helicopter, mobile stroke unit

^b^ Manchester Triage System/Emergency Severity Index categories 1–3

^c^ Manchester Triage System/Emergency Severity Index categories 4–5

Note: Valid percentages are reported, i.e., percentages were calculated excluding missing values. Due to rounding percentages may not always add up to 100

**Fig. 2:**
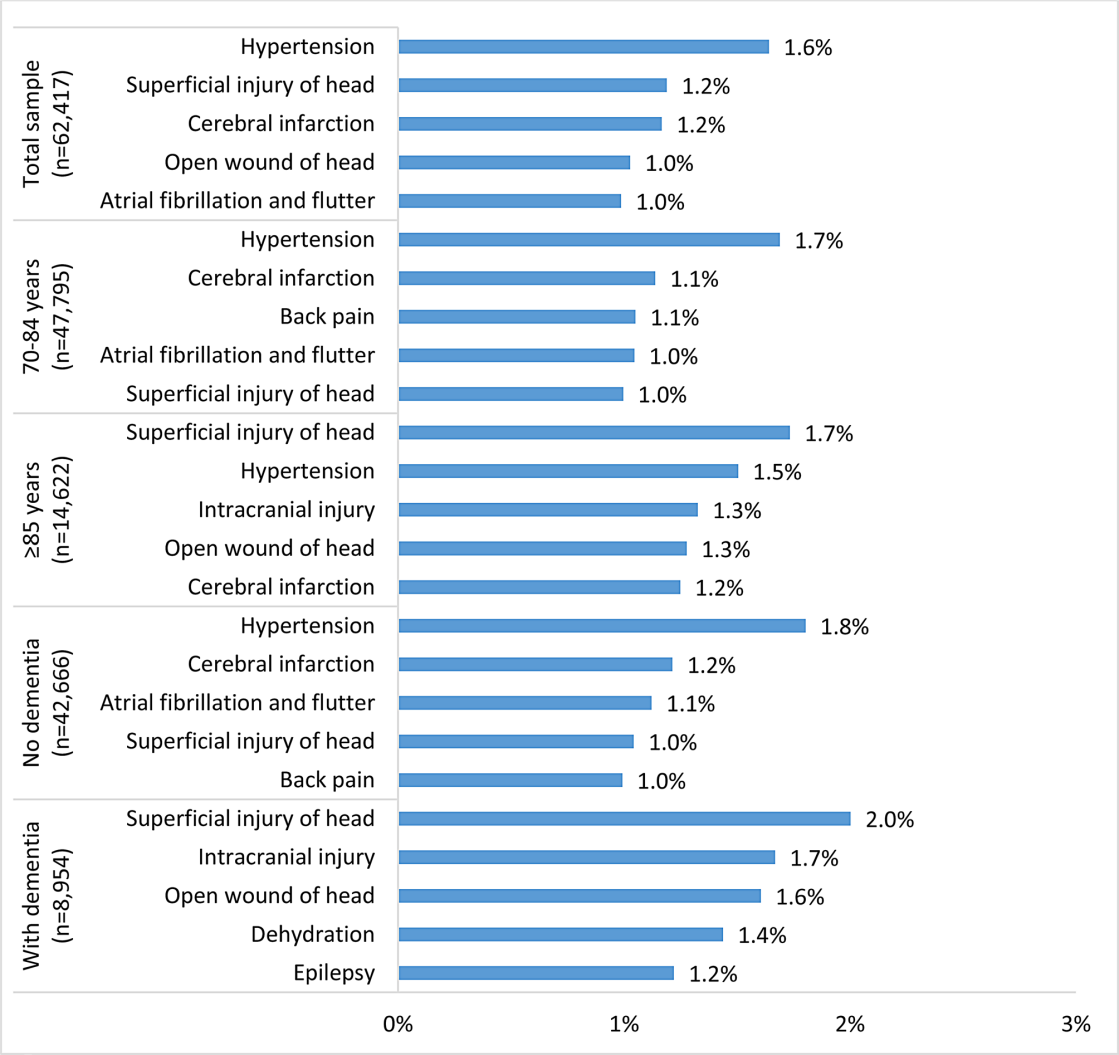
Five most frequent emergency department (ED) diagnoses in the ED population at the index ED visit in 2016 across subgroups* * Missing values in the total sample, *n* = 34,064

ED diagnoses varied between age groups and patients living with and without dementia (Fig. 2). In older patients, injuries of the head and intracranial injuries were the most frequent diagnoses, whilst younger patients presented more often with cardiovascular complaints and back pain.

A main hospital diagnosis was documented for 63,771 patients (63.9%). The five most common main hospital diagnoses were cerebral infarction (7.4%), heart failure (4.4%), intracranial injury (3.5%), acute myocardial infarction (2.8%) and fracture of femur (2.8%) (Fig. S2). Main hospital diagnoses were similar across age subgroups and between patients with and without dementia except for pneumonia, which occurred more often in patients ≥85 years and in patients with dementia with 2.6% and 3.4%, respectively.

### Comorbidity burden and prior outpatient diagnoses

One-third of the outpatient diagnosis population had no comorbidities according to CCI analyses, whereas 36.9%, 20.1% and 10.1% had a CCI of 1–2, 3–4 and ≥5, respectively (Table 2). As dementia is one of the comorbidities accounted for in the CCI, no patient with dementia had a CCI of 0. More patients with dementia had a CCI of 3–4 and ≥5 with 37.5% and 26.6%, respectively, compared to 16.4% and 6.6% in patients without dementia.

**Fig. 3:**
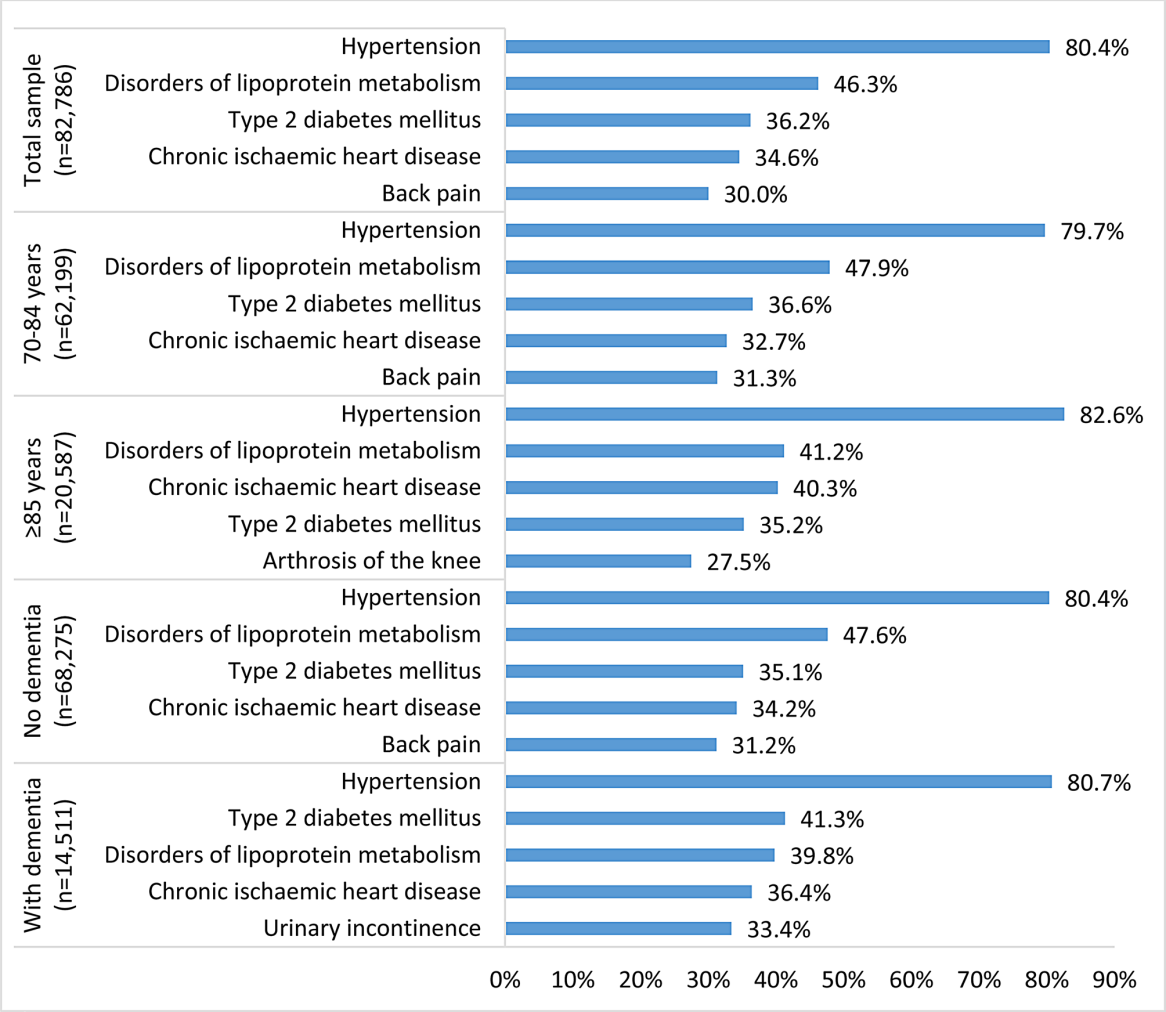
Five most frequent diagnoses of chronic diseases in health claims data in the four quarters (= a period of 12 months) before the index ED visit in 2016 across subgroups*. * Missing values in the total outpatient sample, *n* = 17,072

The five most common chronic outpatient diagnoses were hypertension (80.4%), disorders of the lipoprotein metabolism and other lipidaemias (46.3%), type 2 diabetes mellitus (36.2%), chronic ischaemic heart disease (34.6%) and back pain (30.0%) (Fig. 3). The most frequent chronic diagnoses were similar across the age sub-populations except for individuals ≥85 years, where arthrosis of the knee was the fifth most common disease. In patients with dementia, urinary incontinence was among the five most frequent chronic diagnoses.

### Outpatient care utilization before the ED visit

#### Outpatient physician visits

A total of 84,265 (97.8%) patients of the outpatient population had at least one documented physician (GP or specialist) visit and 82,349 (95.6%) had at least one GP visit within the four quarters preceding the index ED visit (Table 2). A regular GP contact, i.e., a GP visit at least once in all four quarters, was observed for 72,400 (84.1%) ED patients. More ED patients ≥85 years and more ED patients with dementia had regular GP contact compared to the overall outpatient population. A large percentage of the outpatient population (90.7%) also presented at least once to a specialist in the four quarters before the index ED visit. The five most frequently visited specialists were ophthalmologists; orthopaedists; urologists; ear, nose, throat (ENT) doctors; and dermatologists. Among patients with dementia, neurologists were the fifth most visited specialists whereas orthopaedists were not among the top five. For comparison, in patients without dementia, presentations to neurologists were in 12th place.

**Table 2:** Outpatient care utilization and characteristics within the four billing quarters preceding the index ED visit across populations

Outpatient care utilization/ characteristic	Outpatient population *n* = 86,117	70–84 years *n* = 64,978		≥ 85 years *n* = 21,139		Chronic diagnosis population *n* = 82,786	No dementia *n* = 68,275	With dementia *n* = 14,511
Geriatric assessment conducted by the GP within the four billing quarters preceding the ED visit^a^								
Any geriatric assessment, n (%)	39,341 (45.7%)		26,802 (41.2%)		12,539 (59.3%)	39,205 (47.4%)	28,764 (42.1%)	10,441 (71.9%)
No geriatric assessment, n (%)	46,776 (54.3%)		38,176 (58.8%)		8,600 (40.7%)	43,584 (52.6%)	39,511 (57.9%)	4,073 (28.1%)
Basic geriatric assessment (GOP 03360), n (%)	35,551 (41.3%)		24,292 (37.4%)		11,259 (53.3%)	35,435 (42.8%)	26,070 (38.2%)	9,365 (64.5%)
Extended geriatric assessment (GOP 30984), n (%)	10 (0.01%)		7 (0.01%)		3 (0.01%)	10 (0.01%)	7 (0.01%)	3 (0.01%)
Further geriatric care services (GOP 03362), n (%)	36,624 (42.5%)		24,637 (37.9%)		11,987 (56.7%)	34,519 (41.7%)	24,438 (35.8%)	10,081 (69.5%)
Doctor visits within the four billing quarters preceding the ED visit^a^								
≥ 1 doctor visit, n (%)	84,265 (97.8%)		63,451 (97.6%)		20,814 (98.5%)	82,761 (100%)	68,251 (100%)	14,510 (100%)
≥ 1 GP visit, n (%)	82,349 (95.6%)		61,827 (95.2%)		20,522 (97.1%)	81,438 (98.4%)	67,002 (98.1%)	14,436 (99.5%)
Regular GP visits, n (%)	72,400 (84.1%)		53,356 (82.1%)		19,044 (90.1%)	72,391 (87.4%)	58,781 (86.1%)	13,610 (93.8%)
Comorbidity burden according to Charlson Comorbidity Index (CCI)^b^								
No comorbidity, n (%)	27,246 (32.9%)		22,210 (35.7%)		5,036 (24.5%)	27,246 (32.9%)	27,246 (39.9%)	NA
CCI 1–2, n (%)	30,508 (36.8%)		22,935 (36.9%)		7,573 (36.8%)	30,508 (36.9%)	25,298 (37.0%)	5,210 (35.9%)
CCI 3–4, n (%)	16,641 (20.1%)		11,289 (18.1%)		5,352 (26.0%)	16,641 (20.1%)	11,195 (16.4%)	5,446 (37.5%)
CCI ≥ 5, n (%)	8,391 (10.1%)		5,765 (9.3%)		2,626 (12.8%)	8,391 (10.1%)	4,536 (6.6%)	3,855 (26.6%)
Missing values, n	3,331		2,779		552	NA	NA	NA
Prescription medication within the four billing quarters preceding the ED visit^c^								
Polypharmacy, n (%)	37,733 (60.5%)		27,443 (59.6%)		10,290 (63.2%)	40,675 (66.4%)	29,610 (59.0%)	11,065 (68.5%)
Patients without prescription data, n	23,779		18,916		4,863	21,529	18,083	3,446

Abbreviations: CCI, Charlson Comorbidity Index; GOP, billing code of outpatient service (“Gebührenordnungsposition”); GP, general practitioner; NA, not applicable

^a^ Missing values for the variables “geriatric assessment” and “doctor visit” in the overall outpatient population: *n* = 13,741

^b^ For this category, valid percentages are reported, i.e., percentages were calculated excluding missing values. Missing values for the variable “CCI” in the overall outpatient population: *n* = 17,072

^c^ Missing values for the variable “prescription medication” in the overall outpatient population: *n* = 37,520

Note: Due to rounding percentages may not always add up to 100%

#### Patients who had geriatric assessment before the ED visit

Outpatient geriatric assessments by a GP were more often conducted in patients aged ≥85 years with 59.3% (*n* = 12,539) compared with patients 70–84 years with 41.2% (*n* = 26,802) (Table 2). In patients with dementia, geriatric screening was performed in more than two-thirds (71.9%; *n* = 10,441) compared with 42.1% (*n* = 28,764) in patients without dementia. Whilst extended geriatric assessment (GOP 30984) was barely used, there was a roughly equal use of basic assessment (GOP 33060) and subsequent further geriatric care services (GOP 33062).

#### Prescription medications

There were only slight differences in the ranking of the ten most frequently prescribed medications across the different outpatient sub-populations (Table S3). More than half (60.5%) of the patients received ≥5 prescription medications (polypharmacy). In patients with dementia, polypharmacy was observed in more than two-thirds (68.5%) compared with 59.0% in patients without dementia.

### Inpatient mortality of patients with dementia vs. without dementia

A generalized linear mixed model (GLMM) was fitted, adjusted for confounders and study centre as random effect. In the complete case analysis, a total of 64,154 patients were included. The results showed that ED patients with dementia had 18% higher odds of dying in the hospital compared with patients without dementia (OR, 1.18; 95%-CI, 1.08–1.28; p-value < 0.001) when adjusted for sex, age, hypertension, diabetes, atrial fibrillation, depression, obesity and atherosclerosis. For comparison, the unadjusted model resulted in an OR of 1.38 (95% CI, 1.27–1.49; p-value < 0.001).

## Discussion

In our study, 28.2% of ED patients were aged ≥70 years and 14.5% of these older patients had a confirmed diagnosis of dementia when presenting to the ED. We compared the inpatient mortality of patients with dementia vs. patients without dementia using a GLMM and found that ED patients with dementia had 18% higher odds of dying in the hospital subsequent to an ED visit compared with patients without dementia.

The comorbidity burden of our study population was high, with more than two-thirds (67.9%) presenting with a CCI ≥1. Outpatient geriatric care services in the year before the ED visit were conducted in 37.9% of patients aged 70–84 years and amounted to 56.7% and 69.5% in patients aged ≥85 years and patients with dementia, respectively. These findings showed that age-related morbidities and conditions like mobility disorders, cognitive impairment, or frailty were common in the older ED population and underscore postulations that ED staff needs to be trained in geriatric emergency care to provide adequate and age-specific emergency care for older individuals [5, 19, 20]. Our analyses also showed that 40.7% of patients aged ≥85 years and 28.1% of patients with dementia did not have any geriatric assessment in the year before the ED visit, which could indicate an unmet need of geriatric care in the outpatient sector. This assumption is underlined by the large number of ED patients receiving polypharmacy in the very old population and patients living with dementia.

Unfortunately, frailty was no variable in the dataset. It is well known that frailty is highly prevalent in older ED patients and that frailty is associated with mortality after acute ED admission of affected persons as well as with in-hospital complications such as falls, delirium, and hospital-acquired infections [21, 22]. 

Chronic conditions were slightly overrepresented in the study population in comparison to the general German population (hypertension 80.4% vs. 76.0%; Type 2 diabetes mellitus 36.2% vs. 31.0%) [23, 24]. However, it is not surprising that the population seeking help at the ED was sicker than the general population. The high percentage of patients with polypharmacy (60.5%) was consistent with the comorbidity burden of the population, and the most frequently prescribed medications were reflective of the most common chronic diagnoses, which internally validates our findings.

The majority of the ED patients (60.5%) arrived at the ED with medical transportation. In the age group ≥85 years and in patients with dementia, the proportion of medical transportation was even higher in our population, with 75.3% and 81.7%, respectively, while the triage urgency level was comparably high. This indicates that the mobility of patients decreases with increasing age and even more so in patients with dementia. Other studies published similar findings of emergency transport services being more frequently used by individuals of older age and individuals with two or more comorbidities versus individuals with one comorbidity or without chronic disease [25–27]. Naturally, patients needing emergency care may be sicker than the general population and therefore, more in need of medical transportation.

Another explanation for the high use of emergency transportation are structural deficits of the German healthcare system for patients with low mobility as transportation to the GP or other outpatient practices is not reimbursed by the German SHI [28]. Consequently, individuals with low mobility and an acute medical condition might inevitably call the emergency ambulance to get medical attention and present to the ED rather than to a primary care practice as they lack suitable alternative options. This hypothesis was confirmed by a representative telephone survey study by Dahmen et al. (2021), where 37% of the respondents stated that they would call the emergency services in acute medical but not life-threatening situations [29].

A finding from our study pointing in a similar direction was the frequent ED and main hospital diagnoses of hypertension, heart failure, back pain, pneumonia, or dehydration, suggesting that many individuals presented to the ED with ambulatory care–sensitive conditions (ACSC), i.e. conditions which could be managed in the primary care setting and for which ED visits and hospitalizations could be avoided [30, 31]. In addition, almost one-third (31.2%) of the ED visits in our sample were categorised as less urgent. At the same time, it is striking that the large majority of the ED patients had a regular GP contact, i.e., 82.1%, 90.1% and 93.8% of individuals aged 70–84 years, ≥85 years and individuals with dementia, respectively. These contradictory findings on frequent outpatient care utilization versus a high rate of ACSC and non-urgent conditions in the ED could point to potential shortcomings in the quality of healthcare provision in the primary care sector but need to be interpreted including system- and patient-inherent drivers of ED utilization. Additionally, a gap in emergency acute care provision in the primary care sector and a lack of secondary prevention strategies for chronic conditions may lead to the high occurrence [32]. Although ACSCs are used in research and policy making as an indicator to assess the quality of primary healthcare delivery, they are no direct quality measure. In Germany, the high rates of ACSCs in the EDs have been well-known for years [32, 33], and a legislative draft of the German government from July 2024 for a reform of emergency acute care aims to address this outpatient emergency acute care gap [34]. Among others, the reform envisions the implementation of a 24/7 available telemedical consultation and 24/7 medical home visits specifically for patients with mobility issues to ensure the availability of emergency acute care services and to optimize resource use by directing patients as appropriate and preferably to the outpatient care sector [34].

The GLMM showed a statistically significant difference of inpatient mortality in patients with versus without dementia. Whilst the effect size was relatively small, with an OR of 1.18 (95% CI, 1.08–1.28), the effect was consistent with other studies [8, 35]. The increased odds of mortality in patients with dementia stress their increased vulnerability and the need to provide them with adequate geriatric emergency care in the ED. The higher odds of mortality in these patients could also point to a lack of end-of life and palliative care in the primary healthcare sector which contributes to the high rate of ACSC in EDs.

### Strengths and limitations

The fundamental strength of the INDEED study was the linkage of routinely collected ED data with outpatient care data from the SHI. To the best of our knowledge, INDEED is currently the only study in Germany with such data linkage and, therefore, provides unique insights into cross-sectoral care and utilization of healthcare services. Another essential strength of our study is the DAG that identified relevant variables to build a causal model for the regression analysis.

Several limitations must be noted. By design, the INDEED dataset does not include ED cases billed through private health insurance or the German Social Accident Insurance. Therefore, all findings are limited to the SHI population. A primary limitation of our study was the number of missing values in routine data. Missing data were handled as missing completely at random in the GLMM, potentially introducing bias to the analyses. Moreover, the reason for ED admission (presenting complaint) was not part of the dataset and ED diagnoses were not weighted to indicate the main treatment-relevant diagnosis. However, the ED diagnoses provided a relevant overview of the most frequent reasons for ED presentations. The M2Q criterion was used to identify chronic conditions and comorbidity burden. However, it may have led to an underestimation of chronic diagnoses in patients whose initial diagnosis occurred in the quarter prior to the ED visit and failed to capture undiagnosed conditions, which is particularly relevant for stratification in the dementia subgroup. In addition, patients with higher outpatient care utilization were more likely to meet the M2Q criterion, introducing potential ascertainment bias. The INDEED study did not include any variables related to frailty – which is well-known to be associated with mortality [36] – lifestyle or socio-economic factors. Information on the living situation (e.g., living alone or in a nursing home) of our population could have supported the interpretation of the high use of medical transportation to the ED. Furthermore, we could not include relevant confounding variables like frailty, physical (in)activity, smoking status and alcohol consumption into our causal regression model, and the number of unobserved variables limited the interpretation of the model results. Some of the documented GP visits may include visits to solely collect prescriptions for chronic treatments. Finally, a common issue in the analysis of prescription medication is that prescriptions recorded in outpatient care datasets do not necessarily reflect their actual use or treatment adherence. By including only those prescription medications that contained a date of dispensing from the pharmacy, we tried to partially mitigate this issue.

## Conclusion

Older ED patients in Germany presented with a high comorbidity burden, frequently receiving ≥5 medications, and 14.5% were living with dementia. Despite regular contact with the GP prior to the ED visit, we observed a high rate of ACSC and non-urgent conditions in the ED, highlighting how important it is for the German healthcare system to offer adequate alternatives for acute medical conditions, particularly for older individuals. Patients with dementia were more likely to die during the hospital stay following an ED visit compared with patients without dementia. Therefore, it is crucial that EDs build geriatric expertise and are appropriately staffed to address older individuals’ specific emergency care needs. The relationship between dementia and inpatient mortality needs to be further investigated in primary research including relevant clinical and lifestyle variables.

## Supplementary Information

Below is the link to the electronic supplementary material.

Supplementary Material 1

Supplementary Material 2

## Data Availability

Due to data protection legislation in Germany the data of this study are not available for the public. Data availability was restricted to the INDEED consortium.
