# Supplementary material for: Emergency department presentations of older patients in Germany: high rates of ambulatory care–sensitive conditions and increased odds of inpatient mortality in patients living with dementia
Source: BMC Emerg Med. 2026 Jan 6;26:31. doi: 10.1186/s12873-025-01458-8 (PMC12849080; doi:10.1186/s12873-025-01458-8)
Supplement: Supplementary file 1 — Supplementary Material 1 [file 12873_2025_1458_MOESM1_ESM.docx]

# Definition of descriptive variables

**Table S1** Definition of ED and outpatient descriptive variables

| Variable | Definition | Missing values, n (%) | | |
| --- | --- | --- | --- | --- |
|  |  | Cases | | Patients |
| Age | Continuous variable defined as the age of the case/individual patient at the time of the ED visit. A categorical variable was created for the age sub-populations of *≥70 years*, *70-84 years* and *≥85 years*.  A patient who visited the ED more than once could present with an older age at the next visit and thus, could have two different age entries in the dataset. Therefore, for the patient-based analysis, patients were grouped based on the age of their first (index) ED visit. | None | | None |
| Sex | Binary variable defined as either *female* or *male*. | None | | None |
| Transport to ED | Categorical variable that describes how a case/individual patient arrived at the ED. Options are:   - *By own means* - *Medically accompanied* (ambulance, emergency ambulance, intensive care transport vehicle/helicopter, rescue helicopter, mobile stroke unit) - *Other* | 39,280 (28.3%) | | 28,872 (28.9%) |
| Triage category | Two different systems of triage categorization were used across the 16 EDs of the INDEED project, i.e., the Manchester Triage System (MTS) or the Emergency Severity Index (ESI). Triage categories ranged from 1 to 5 with 1 being the most urgent and 5 being non-urgent. For this analysis, the categories were summarized into a binary variable as follows:   - *Urgent* summarised the categories 1 (“resuscitation”), 2 (“emergent”) and 3 (“urgent”) - *Less urgent* summarised categories 4 (“less urgent”) and 5 (“non-urgent”) | 27,761 (20.0%) | | 20,535 (20.6%) |
| ED admission within working hours | Binary variable that defines whether a case/individual patient presented to the ED *within hours*, i.e., during a workday AND during core working hours (7:00 am – 18:59 pm) or whether the case/individual presented to the ED *out of hours*, i.e., on the weekend, a public holiday AND/OR outside the core working hours (19:00 pm – 6:59 am). | 671 (0.5%) | | 301 (0.3%) |
| Day of ED admission | Binary variable that defines whether a case/individual patient presented to the ED on a *weekday* vs. on a *weekend/public holiday*. | 671 (0.5%) | | 301 (0.3%) |
| Season | Categorical variable which defines during which season (*spring*, *summer*, *autumn* or *winter*) a case/individual patient presented to the ED. | 671 (0.5%) | | 301 (0.3%) |
| Type of hospital stay | Binary variable that describes whether the case/individual patient was treated as *ambulatory* case or admitted to the hospital and treated as *inpatient* case. | None | | None |
| Death | Binary variable that describes whether the case/individual patient died in the hospital or not. | 29,447 (21.2%) | | 20,872 (20.9%) |
| Frequency of ED admission | Ordinal variable that describes how often an individual patient was admitted to the ED per year, i.e., *1-2x per year*, *3-9x per year* or *more than 9x per year*. This variable was used for analyses based on the individual patient only and not for any analyses based on ED cases. | None | | None |
| ED diagnoses | Categorical variable based on ICD-10 coding system of diagnoses. One ED case/individual patient could have several ED diagnoses. ED diagnoses were available for 13 out of 16 EDs. One of the 13 EDs documented extraordinary many ED diagnoses per case which strongly influenced the ranking of the 10 most common ED diagnoses. Therefore, it was decided to exclude this centre from the analyses as it biased the results. | 85,137  (61.4%) | | 62,417 (62.5%) |
| Main hospital diagnosis | Categorical variable based on ICD-10 coding system of diagnoses which was assigned to cases/individual patients who were hospitalised following the ED visit. | 81,740 (37.3%) | | 36,087 (37.1%) |
| Geriatric assessment | In the German healthcare system, a geriatric assessment of a patient can be conducted if the patient is at least 70 years of age and among others, presents with typical geriatric morbidities like mobility disorders, cognitive impairment, frailty syndrome, incontinence or dysphagia. The geriatric assessment is recorded and billable according to outpatient services billing codes (“Gebührenordnungsposition”, GOP) of the SHI doctor’s fee scale (“Einheitlicher Bewertungsmaßstab” [uniform value scale], EBM). GOP codes were available for a total of 86,117 patients ≥70 years who presented to the ED in 2016 (outpatient population). Of note, data from one ED was excluded from the outpatient population as this centre provided GOP data for only 11 ED patients.  GOP 03360 is used for the billing of basic geriatric assessment, GOP 30984 for extended geriatric assessment and GOP 03362 for further geriatric care services by the GP. The latter can only be billed if a prior geriatric assessment based on GOP 03360 or 30984 was conducted.  For this research GOP codes 03360 and 30984 were used to assess outpatient care services and quality in the older patient population. GOP code 03362 was used to assess the prevalence of frailty and age-related morbidities in the outpatient population.  ED patients who had at least one geriatric assessment within the four billing quarters preceding the index ED visit were identified and a binary variable was created indicating whether a patient had any type of *geriatric assessment yes/no*. In addition, categorical variables were created to assess whether a patient had a *basic geriatric assessment (GOP 03360)*, *an extended geriatric assessment (GOP 30984)* or *further geriatric care services by the GP (GOP 03362)*. | NA | 13,741  (13.8%) | |
| GP/specialist visits | GP/specialist visits (categorical variable) were identified via outpatient billing data (GOP codes). In the German SHI system, outpatient services are recorded and billed via GOP codes which also specify the type of physician who provided the outpatient service based on a GOP code. The analysis of GP/specialist visits was limited to the four billing quarters preceding the index ED visit.  GOP codes were available for a total of 86,117 patients ≥70 years who presented to the ED in 2016 (outpatient population). Of note, data from one ED was excluded from the outpatient population as this centre provided GOP data for only 11 ED patients. | NA | 13,741  (13.8%) | |
| Charlson Comorbidity Index (CCI) | Using weights, the CCI is a validated and commonly used score to predict mortality and to measure the burden of disease of an individual based on their comorbidities. The original score and weights were developed by Charlson et al. and published in 1987.[2] In 2011, Quan et al. published an updated, validated version of the CCI, taking into account that the weights attributed to individual comorbidities have changed due to advances in medicine.[3] In this research, we applied the updated weights of the CCI by Quan et al. (2011).[3]  Diagnoses were recorded as “confirmed”, “suspected”, “past” or “excluded” in the dataset and only “confirmed” diagnoses were included in our study. Moreover, chronic diagnoses/comorbidities were defined according to the M2Q criteria. This means that the diagnosis of a comorbidity had to be documented in two or more quarters within the four billing quarters preceding the index ED visit. If a comorbidity of an individual was present in different degrees of severity, only the more severe form was included in the score.  Data on outpatient chronic diagnoses were available for a total of 82,786 patients aged ≥70 years who presented to the ED in 2016 and who had GOP data available (chronic diagnosis population).  CCI was categorised as follows: *CCI 0*, *CCI 1-2*, *CCI 3-4*, and *CCI ≥5*. | NA | 17,072  (17.1%) | |
| Prior diagnoses | Categorical variable based on ICD-10 coding system of diagnoses as documented in the SHI records. Outpatient diagnoses were recorded as “confirmed”, “suspected”, “past” or “excluded” in the dataset and only “confirmed” diagnoses were included in our study. Moreover, chronic diagnoses were defined according to the M2Q criteria. This means that the diagnosis of a disease had to be documented in two or more quarters within the four billing quarters preceding the index ED visit. Data on outpatient chronic diagnoses were available for a total of 82,786 patients aged ≥70 years who presented to the ED in 2016 and who had GOP data available (chronic diagnosis population). | NA | 17,072  (17.1%) | |
| Dementia | Binary variable indicating whether an individual had a diagnosis of *dementia or not* according to ICD-10 codes F00, F01, F02, F03, or G30 applying the M2Q criterion. Diagnoses were recorded as “confirmed”, “suspected”, “past” or “excluded” in the dataset and only “confirmed” diagnoses were included. M2Q means that the diagnosis of dementia had to be documented in two or more quarters within the four billing quarters preceding the index ED visit. Data on outpatient chronic diagnoses were available for a total of 82,786 patients aged ≥70 years who presented to the ED in 2016 and who had GOP data available (outpatient diagnosis population). | NA | 17,072  (17.1%) | |
| Prescription medications | Medications prescribed by a physician are documented in the SHI records based on Anatomical Therapeutic Chemical Classification (ATC codes). ATC codes were used to identify the most frequently prescribed medications within the two quarters prior to the index ED visit. Polypharmacy was defined as taking five or more medications and a binary variable *polypharmacy yes/no* was created.  Four ED centres did not transmit complete prescription medication data and were therefore excluded from the analysis. In total, data on prescription medications were available for 62,338 patients ≥70 years who presented to the ED in 2016 and who had GOP data available. | NA | 37,520  (37.6%) | |

Abbreviations: ATC, Anatomical Therapeutic Chemical Classification; EBM, Einheitlicher Bewertungsmaßstab (uniform value scale); ED, emergency department; GOP, Gebührenordnungsposition (outpatient services billing code); GP, general practitioner; ICD, International Statistical Classification of Diseases and Related Health Problems; INDEED, utilization and cross-sectoral patterns of care for patients admitted to emergency departments in Germany; M2Q, two or more quarters; NA, not applicable; SHI, statutory health insurance.

# Demographic and clinical characteristics of ED cases

**Table S2** Demographic and clinical characteristics of ED cases

| Characteristic | Total sample  n=138,652 | 70-84 years  n=103,637 | ≥85 years  n=35,015 |
| --- | --- | --- | --- |
| Age, median (IQR) | 79 (75, 85) | 77 (74, 80) | 88 (86, 91) |
| Female, n (%) | 76,215 (55%) | 53,004 (51.1%) | 23,211 (66.3%) |
| Transport to ED | | | |
| By own means, n (%) | 30,559 (30.8%) | 25,862 (34.9%) | 4,697 (18.6%) |
| Medically accompanied^a^, n (%) | 61,221 (61.6%) | 42,216 (56.9%) | 19,005 (75.4%) |
| Other, n (%) | 7,592 (7.6%) | 6,086 (8.2%) | 1,506 (6%) |
| Missing values (n) | 39,280 | 29,473 | 9,807 |
| Triage category | | | |
| Urgent^b^, n (%) | 73,806 (66.6%) | 55,293 (66.5%) | 18,513 (66.8%) |
| Less urgent^c^, n (%) | 37,085 (33.4%) | 27,876 (33.5%) | 9,209 (33.2%) |
| Missing values, n | 27,761 | 20,468 | 7,293 |
| Time of ED admission | | | |
| 7:00 am – 18:59 pm, n (%) | 75,924 (55%) | 56,894 (55.2%) | 19,030 (54.6%) |
| 19:00 pm – 6:59 am, n (%) | 62,057 (45%) | 46,247 (44.8%) | 15,810 (45.4%) |
| Missing values, n | 671 | 496 | 175 |
| Day of ED admission | | | |
| Weekday, n (%) | 99,406 (72%) | 74,337 (72.1%) | 25,069 (72%) |
| Weekend/Public holiday, n (%) | 38,575 (28%) | 28,804 (27.9%) | 9,771 (28%) |
| Missing | 671 | 496 | 175 |
| Season of ED admission | | | |
| Spring, n (%) | 35,599 (25.8%) | 26,666 (25.9%) | 8,933 (25.6%) |
| Summer, n (%) | 34,083 (24.7%) | 25,756 (25%) | 8,327 (23.9%) |
| Autumn, n (%) | 33,672 (24.4%) | 24,983 (24.2%) | 8,689 (24.9%) |
| Winter, n (%) | 34,627 (25.1%) | 25,736 (25%) | 8,891 (25.5%) |
| Missing values, n | 671 | 496 | 175 |
| Type of admission | | | |
| Ambulatory, n (%) | 51,077 (36.8%) | 39,222 (37.8%) | 11,855 (33.9%) |
| Inpatient, n (%) | 87,575 (63.2%) | 64,415 (62.2%) | 23,160 (66.1%) |
| Death | | | |
| Inpatient death, n (%) | 5,876.0 (5.4%) | 3,709.0 (4.6%) | 2,167.0 (7.6%) |
| Missing values, n | 29,447 | 22,776 | 6,671 |

Abbreviations: ED, emergency department; IQR, interquartile range.

^a^ ambulance, emergency ambulance, intensive care transport vehicle/helicopter, rescue helicopter, mobile stroke unit

^b^ Manchester Triage System/Emergency Severity Index categories 1-3.

^c^ Manchester Triage System/Emergency Severity Index categories 4-5.

Note: Valid percentages are reported, i.e., percentages were calculated excluding missing values. Due to rounding percentages may not always add up to 100%.

# Most frequently prescribed medications

**Table S3** Ten most frequently prescribed medications across populations in the two quarters prior to the index ED visit

| **Medication** | **N** | **%** |
| --- | --- | --- |
| Total sample (n=62,338) | | |
| Pantoprazole | 31,712 | 50.9% |
| Torasemide | 25,663 | 41.2% |
| Ramipril | 24,077 | 38.6% |
| Simvastatin | 24,028 | 38.5% |
| Metoprolol | 23,901 | 38.3% |
| Metamizole sodium | 22,770 | 36.5% |
| Bisoprolol | 21,773 | 34.9% |
| Amlodipine | 18,987 | 30.5% |
| Acetylsalicylic acid | 16,576 | 26.6% |
| Levothyroxine sodium | 15,881 | 25.5% |
| Patients aged 70-84 (n=46,062) | | |
| Pantoprazole | 22,778 | 49.5% |
| Simvastatin | 18,425 | 40.0% |
| Metoprolol | 17,315 | 37.6% |
| Ramipril | 17,055 | 37.0% |
| Torasemide | 16,441 | 35.7% |
| Bisoprolol | 15,853 | 34.4% |
| Metamizole sodium | 15,387 | 33.4% |
| Amlodipine | 13,765 | 29.9% |
| Levothyroxine sodium | 12,158 | 26.4% |
| Acetylsalicylic acid | 11,588 | 25.2% |
| Patients aged ≥85 years (n=17,895) | | |
| Torasemide | 9,222 | 56.7% |
| Pantoprazole | 8,934 | 54.9% |
| Metamizole sodium | 7,383 | 45.4% |
| Ramipril | 7,022 | 43.1% |
| Metoprolol | 6,586 | 40.5% |
| Bisoprolol | 5,920 | 36.4% |
| Simvastatin | 5,603 | 34.4% |
| Amlodipine | 5,222 | 32.1% |
| Acetylsalicylic acid | 4,988 | 30.6% |
| Levothyroxine sodium | 3,723 | 22.9% |
| Patients without dementia (n=50,192) | | |
| Pantoprazole | 24,686 | 49.2% |
| Torasemide | 19,481 | 38.8% |
| Simvastatin | 19,363 | 38.6% |
| Metoprolol | 19,164 | 38.2% |
| Ramipril | 18,726 | 37.3% |
| Bisoprolol | 17,914 | 35.7% |
| Metamizole sodium | 17,472 | 34.8% |
| Amlodipine | 15,386 | 30.7% |
| Levothyroxine sodium | 13,111 | 26.1% |
| Acetylsalicylic acid | 12,400 | 24.7% |
| Patients with dementia (n=11,065) | | |
| Pantoprazole | 6,575 | 59.4% |
| Torasemide | 5,803 | 52.4% |
| Ramipril | 4,973 | 44.9% |
| Metamizole sodium | 4,948 | 44.7% |
| Metoprolol | 4,352 | 39.3% |
| Simvastatin | 4,321 | 39.1% |
| Acetylsalicylic acid | 3,986 | 36.0% |
| Bisoprolol | 3,569 | 32.3% |
| Amlodipine | 3,337 | 30.2% |
| Glucose, test zone blood | 2,578 | 23.3% |

# Most frequent main hospital diagnoses

**Figure S1** Five most frequent main hospital diagnoses at the index ED visit across population
